# Comparison of volumes of brain areas in patients with bilateral early high-tension and normal-tension glaucoma in 7 Tesla MRI

**DOI:** 10.1371/journal.pone.0341306

**Published:** 2026-01-23

**Authors:** Sylwester Matwiejczuk, Anna Niedziałek, Katarzyna Toborek, Mateusz Midura, Dominika Wróbel-Dudzińska, Tomasz Żarnowski, Radosław Pietura, Ewa Kosior-Jarecka

**Affiliations:** 1 Department of Diagnostics and Microsurgery of Glaucoma, Medical University of Lublin, Lublin, Poland; 2 Department of Radiography, Medical University of Lublin, Lublin, Poland; 3 Center for Artificial Intelligence and Computer Modeling, Maria Curie-Skłodowska University, Lublin, Poland; 4 Faculty of Electronics and Information Technology, Institute of Radioelectronics and Multimedia Technology, Warsaw University of Technology, Warszawa, Poland; Seirei Hamamatsu General Hospital, JAPAN

## Abstract

**Background:**

Glaucoma is an optic neuropathy characterized by progressive retinal ganglion cells degeneration and associated visual field defects. Although elevated intraocular pressure is a major risk factor, glaucoma can also develop in individuals with statistically normal intraocular pressure, implying alternative mechanisms such as neurotrophins deprivation and primary lateral geniculate nucleus injury. These mechanisms may suggest a neurodegenerative aspect of the disease.

**Methods:**

In this study, we evaluated 15 patients with bilateral early-stage normal-tension glaucoma, 10 with bilateral early-stage high-tension glaucoma, and 15 age-matched controls. All participants underwent standard ophthalmic examinations, including visual field testing and retinal nerve fiber layer assessment. High-resolution brain imaging was performed on a 7 Tesla MRI scanner. Volumes of the lateral geniculate nucleus and additional brain structures were quantified using ITK-SNAP and FreeSurfer.

**Results:**

Lateral geniculate nucleus volumes were significantly reduced in both normal-tension glaucoma and high-tension glaucoma groups compared to controls for left side, with no significant difference between normal-tension glaucoma and high-tension glaucoma. Average lateral geniculate nucleus volume was significantly lower in the normal-tension glaucoma group relative to controls, but not in high-tension glaucoma. Pearson correlation showed moderate positive associations between lateral geniculate nucleus volume and average retinal nerve fiber layer thickness when all participants were analyzed together, but no significant correlations were observed within individual groups. No significant differences in total brain volume or cortical structures (e.g., primary and secondary visual cortices) were observed among the three groups. Although certain non-visual regions, such as entorhinal cortex and fusiform gyrus, exhibited subtle alterations suggestive of early neurodegenerative processes, these differences did not reach statistical significance. Nevertheless, these findings may justify further investigation in larger cohorts to explore their potential as early indicators of neurodegeneration in glaucoma.

**Conclusion:**

These findings indicate that the impact of glaucoma might not be limited to the optic nerve but could also involve alterations in central visual and non-visual pathways. The use of 7 Tesla MRI showed promise in detecting early structural changes, highlighting the importance of further developing advanced imaging techniques. This could help expand the clinical use of ultra-high-field MRI, enhancing diagnosis and understanding of glaucoma.

## 1. Introduction

Glaucoma refers to a group of optic neuropathies characterized by the progressive degeneration of retinal ganglion cells (RGCs). This degeneration leads to optic disc cupping, a distinct structural change and visual loss, which manifests as scotomas in visual field (VF) examinations [1].

Glaucoma affects more than 70 million people worldwide, with 10% experiencing bilateral blindness [2], making it the leading cause of irreversible blindness globally. Glaucomatous optic neuropathy (GON) remains asymptomatic until its advanced stages, suggesting that the actual number of affected individuals may be significantly higher [3]. Glaucoma is broadly classified into two categories: open-angle glaucoma and angle-closure glaucoma. In Europe, over 80% of cases fall into the open-angle glaucoma category [4].

The biological basis of glaucoma remains unclear, and the factors contributing to its development and progression have not been fully characterized [1]. Elevated intraocular pressure (IOP) is the only modifiable ocular risk factor in the pathogenesis of GON, all therapies with proven efficacy in slowing glaucoma progression are based on IOP reduction. However, only a small proportion of individuals with elevated IOP develop glaucoma, while a significant number of glaucoma patients have never experienced IOP levels exceeding the statistical norm. The correlation between IOP levels and glaucoma progression is weak [5].

GON can develop even in individuals with normal IOP in condition known as normal-tension glaucoma (NTG). In such cases, factors other than elevated IOP are believed to contribute to disease progression. One proposed mechanism is impaired microcirculation, leading to insufficient blood supply to the optic nerve. Additionally, an abnormally low cerebrospinal fluid pressure in the optic nerve subarachnoid space may create a significant pressure gradient across the lamina cribrosa [6,7]. Moreover, processes such as excitotoxicity, immune system dysfunction, and excessive oxidative stress may also play a role in the development of glaucomatous neuropathy [1].

Another hypothesis suggests that primary neural pathological processes may trigger secondary neurodegeneration, affecting other retinal neurons and cells in the central visual pathway by altering their environment and increasing their vulnerability to damage [8]. Some studies have also indicated a potential link between glaucoma and neurodegenerative diseases, particularly Alzheimer’s disease (AD) and Parkinson’s disease [9–11].

Neurotrophins regulate neuronal growth, function and survival. RGCs receive neurotrophins from Müller cells [12] or via retrograde transport from the brain. In glaucoma, this transport is blocked at the optic nerve head, depriving RGCs of brain-derived neurotrophic factor (BDNF) support from the lateral geniculate nucleus (LGN) [13–15], leading to RGCs apoptosis in glaucoma [16]. While the etiology of glaucoma remains debated, neurotrophins deprivation due to axonal transport failure is a key factor [17]. RGCs are supported by multiple growth factors, with BDNF being the most crucial [18–20]. Neurotrophins loss may result from transport impairment in high-tension glaucoma (HTG) or disrupted production in NTG. Quigley et al. showed acute IOP elevation suppresses retrograde BDNF transport, leading to neuronal loss [13]. BDNF also supports postsynaptic neurons via anterograde transport to the central nervous system [21].

Given that RGC damage may result not only from disrupted neurotrophin transport due to elevated IOP but also from insufficient neurotrophin production secondary to primary LGN injury, these findings support the hypothesis of glaucoma as a neurodegenerative disorder, with the LGN as the primary site of damage. This perspective prompted the present study, which assessed the volumes of the LGN and other brain structures in patients with early-stage NTG and HTG.

**Aim**: The aim of this study was to evaluate and compare both visual and non-visual brain structures in patients with NTG and HTG at the early bilateral stage of the disease. High-resolution imaging allows for a precise assessment of brain structures, offering the potential for a better understanding of the disease’s pathophysiology and the development of a new diagnostic tool, particularly in challenging cases where standard methods are insufficient.

## 2. Materials and methods

### 2.1. Patients

The study was conducted at the Department of Diagnosis and Microsurgery of Glaucoma and the Department of Radiography at the Medical University of Lublin, Poland, with approval from the Local Ethics Committee (approval number KE 0254/201/2021). Participants with glaucoma and healthy controls were recruited between 29 November 2021 and 29 September 2022. Written informed consent was obtained from all individual participants included in the study. All procedures adhered to the principles of the Declaration of Helsinki.

The study cohort included patients with bilateral early-stage NTG (n = 15), bilateral early-stage HTG (n = 10), and an age-matched control group of healthy volunteers (n = 15). Optical coherence tomography (OCT) was performed using the Cirrus HD-OCT 6000 (Carl Zeiss Meditec, Dublin, CA, USA; software version 11.5.2.54532) with automatic segmentation provided by the manufacturer’s software. Peripapillary RNFL (retinal nerve fibre layer) thickness measurements were included only if the signal strength was ≥ 6/10, scans below this threshold were repeated and excluded if poor quality persisted. Standard automated perimetry was performed using the Humphrey Field Analyzer 745i (Carl Zeiss Meditec, software version 5.1.2) with the SITA Fast 24-2 strategy. Patients with ocular or systemic conditions that could affect the optic nerve or visual pathway, including uveitis, optic neuritis, diabetic retinopathy, advanced cataract, age-related macular degeneration, retinal vascular occlusion, previous intraocular surgery (except uncomplicated cataract extraction), or neurological/cerebrovascular disease, were excluded from the study. Patients with high myopia (spherical equivalent ≤ −6.0 diopters) and high hyperopia (spherical equivalent ≥ +5.0 diopters) were excluded from the study [22,23]. All glaucoma patients met the criteria for early-stage disease as defined by the Hodapp-Parrish-Anderson classification, based on results from the 24-2 VF test, with experienced glaucoma specialist verifying the diagnoses. Only those with bilateral symmetry in defect staging were included. A glaucomatous defect on standard automated perimetry was defined as a hemifield test result outside normal limits, with at least three contiguous points in the same hemifield on the pattern deviation plot at p < 1% (including one point at p < 0.5%) on at least two consecutive tests, and reliability indices <15%.

Glaucoma diagnosis criteria included glaucomatous neuroretinal rim thinning, an open angle on gonioscopy, and the absence of ocular pathology suggestive of secondary glaucoma. In the NTG group, IOP remained consistently below 21 mmHg, with maximum IOP evaluated through four office-hour measurements. IOP was recorded on two separate visits, with two measurements per visit spaced by a two-hour interval. For newly diagnosed patients, IOP measurements were obtained before the initiation of any hypotensive therapy, whereas for patients previously treated in other centers, measurements were taken during ongoing treatment, and the highest untreated IOP value from medical history was used for classification. Patients were categorized as HTG if any IOP reading reached 30 mmHg or higher. Diagnoses were confirmed by experienced glaucoma specialist. The control group was recruited from individuals who, following a full ophthalmic assessment, showed no signs of glaucoma or other conditions affecting the visual pathways, such as retinal or optic nerve disorders, or central nervous system pathology.

### 2.2. 7 Tesla (7T) MRI data acquisition

Imaging was performed at the ECO-TECH COMPLEX in Lublin, Poland, using a 7T Discovery MR950 MRI system (GE Healthcare), equipped with a gradient strength of 50 mT/m and a slew rate of 200 T/m/s. A two-channel birdcage coil operated in quadrature mode was used for transmission, while signal reception was carried out with a 32-channel array coil (Nova Head 32-channel head coil, 2Tx/32Rx). The 7T imaging protocol included two sequences: 3D BRAVO T1-weighted and 3D MT-weighted SILENT, acquired using the parameters listed in Table 1.

**Table 1:** Imaging protocols used in the present study for 7T MRI of the brain.

	3D BRAVO T1-weighted	3D MT-weighted SILENT
Sequence duration [min:s]	4:24	6:30
Field Of View [cm]	22 x 22	17.6 x 17.6
Slice thickness [mm]	1.0	0.8
Echo Time [ms]	2.6	0.0
Repetition Time [ms]	6.6	257
Inversion Time [ms]	450	not applicable
Matrix size	288 x 288	224 x 224
Number of Excitations	1	3
Flip Angle [°]	12	2

**Fig 1:**
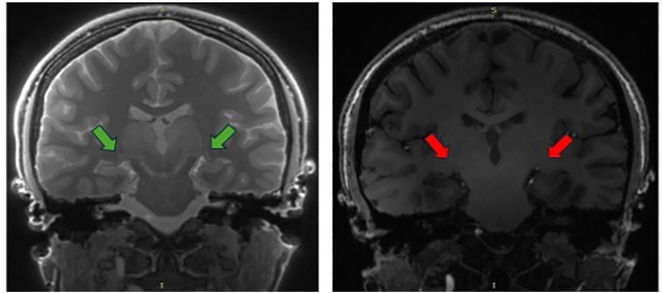
Comparison of LGN visibility (arrows) in two sequences. On the left side is 3D MT-W SILENT; on the right side is 3D T1-W BRAVO. Images acquired at the Ecotech Complex (Lublin, Poland).

The 3D MT-weighted SILENT sequence provided a nearly free of acoustic noise images, with highly effective fat suppression and short acquisition time. Compared to the 3D BRAVO T1-weighted sequence, where the LGN is barely visible and difficult to assess, the 3D MT-W SILENT sequence offered significantly improved visualization of small structures such as the LGN (Fig 1).

### 2.3. Data analysis

Cortical thickness and volumetric analyses were conducted using FreeSurfer (version 7.4.1; Massachusetts General Hospital, Harvard Medical School; http://surfer.nmr.mgh.harvard.edu, accessed on 20 June 2023), an open-source, automated neuroimaging software package. The analysis employed the recon-all, which reconstructs a two-dimensional cortical surface from a high-resolution, three-dimensional T1-weighted anatomical scan-optimally suited for distinguishing grey and white matter due to its high contrast. We used the standard version of the recon-all procedure, which includes preprocessing steps such as skull stripping, intensity normalization, volumetric alignment, segmentation, smoothing, and cortical parcellation (https://surfer.nmr.mgh.harvard.edu/fswiki/recon‐all, accessed on 20 June 2023). The anatomical images were acquired with an isotropic voxel size of 1 mm³. Segmentation outputs were summarized using FreeSurfer’s built-in reporting scripts. For volume quantification, regions were identified based on the Desikan-Killiany cortical parcellation atlas.

The volume of the LGN was measured using ITK-SNAP (version 4.0.0-rc.2), a tool designed for manual segmentation of anatomical regions of interest in 3D medical imaging data. The 3D MT-weighted SILENT sequence was independently assessed by two radiologists. To ensure consistency across measurements, all radiologists applied the same linear contrast settings: minimum intensity 250, maximum 1750, with a window level of 1000 and a window width of 1500. As a result, their volume estimations were closely aligned.

### 2.4. Statistical analysis

All statistical analyses and data visualizations were performed using R software (version 4.3.3; R Core Team). Key packages used included dplyr and tidyr for data manipulation, ggplot2 for visualization, and car and broom for statistical modeling and results interpretation. The statistical significance threshold was set at α = 0.05. Region-wise statistical comparisons were controlled for multiple testing using the false discovery rate (FDR) procedure. OCT and visual field metrics were analyzed at the eye level, with both eyes treated as independent observations, preliminary checks indicated minimal inter-eye correlation (ρ < 0.2), so mixed-effects modeling was not required.

To assess group differences in ophthalmic and brain imaging parameters among the NTG, HTG, and control groups, one-way ANOVA was used where parametric assumptions were met. Tukey’s post hoc test was applied to determine pairwise differences when ANOVA yielded significant results. Normality was assessed using the Shapiro-Wilk test, and homogeneity of variances with Levene’s test.

For interobserver agreement in LGN volume measurements, one-way ANOVA was applied for the right side (assumptions met), and the Kruskal-Wallis test was used for the left side due to non-normality and heterogeneity of variances. The mean of four volume measurements was used in further analyses.

Comparisons of LGN volumes (left, right, average) between groups were conducted with ANOVA. Significant group effects were followed by post hoc tests. For correlation analysis between LGN volume and RNFL parameters, Pearson’s correlation coefficient was calculated. Assumptions for parametric correlation analysis, including normality and linearity, were verified prior to testing. Analyses were performed both within individual groups and across the pooled sample. For each correlation, 95% confidence intervals for r were reported (as shown in Table 8), together with corresponding p-values.

In addition, pairwise comparisons of VFI between NTG and HTG were performed separately for each eye: an independent-samples Student’s t-test was used for the right eye (RE), while the Wilcoxon rank sum test with continuity correction was used for the left eye (LE).

Age, sex, and brain volume were considered as potential covariates. Group comparability with respect to these variables was assessed a priori (Table 1 and Table 8), and no statistically significant differences between groups were observed. In addition, exploratory analyses showed no association between age, sex or brain volume and LGN volume in the present sample. Therefore, these variables were not included as covariates in the primary statistical models, and group comparisons were performed using unadjusted analyses. Unadjusted group estimates with 95% confidence intervals are reported in S1 Table.

## 3. Results

Participant age did not differ significantly between groups, as assessed by one-way ANOVA (p = 0.293). The NTG group was the youngest (M = 64.8 years, SD = 7.8), while the HTG group was the oldest (M = 69.0 years, SD = 6.80). Sex distribution also did not differ significantly between groups (chi-square test, p = 0.083), according to Table 2.

**Table 2:** Demographic characteristics of the studied groups.

Group	n	Age [yrs]: mean ± SD [min – max]	Female n (%)	Male n (%)
**NTG**	15	64.8 ± 7.80 [52 - 78]	14 (93%)	1 (7%)
**HTG**	10	69.0 ± 6.80 [54 - 78]	6 (60%)	4 (40%)
**Control**	15	67.8 ± 6.11 [58 - 78]	12 (80%)	3 (20%)
**p-value**		0.293^*^	0.083^**^	0.083^**^

*p-value for age: one-way ANOVA

**p-value for sex: chi-square test

Clinical characteristics of the studied groups are presented in Table 3. The mean visual field index (VFI) for the right eye (RE) was significantly lower in the NTG group (88.67 ± 7.03%) compared to the HTG group (94.60 ± 3.81%; p = 0.012). Similarly, for the left eye (LE), the mean VFI was also significantly reduced in the NTG group (88.40 ± 8.64%) relative to the HTG group (94.30 ± 3.13%; p = 0.026). These findings indicate a statistically significant deterioration of VF function in both eyes in patients with NTG compared to those with HTG. This finding indicates that, despite comparable MD values, the VFI is significantly lower in the NTG group compared to the HTG group. This may be attributed to the fact that visual field defects in NTG tend to occur closer to the fixation point, leading to a greater impact on the VFI.

**Table 3:** Clinical characteristics of the studied groups.

	NTG				HTG				p-value
	Mean	SD	Min	Max	Mean	SD	Min	Max	
**MD RE [db]**	−3.18	7.03	−6.79	−0.17	−2.83	3.81	−5.36	−0.51	0.657
**MD LE [db]**	−3.71	2.07	−6.97	−0.27	−3.04	1.44	−5.24	−0.63	0.382
**VFI RE (%)**	88.67	7.03	76.00	98.00	94.60	3.81	88.00	98.00	0.012^*^
**VFI LE (%)**	88.40	8.64	74.00	99.00	94.30	3.13	89.00	98.00	0.026^*^

* statistically significant difference (p < 0.05); Student’s t-test

**Fig 2:**
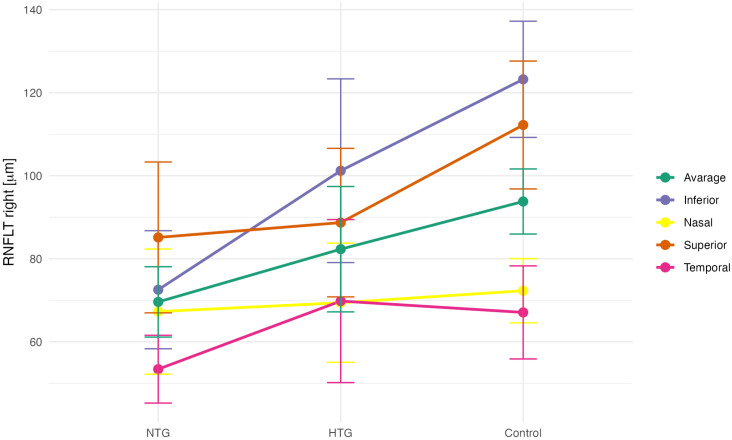
Mean RNFL thickness values (right eye) in the NTG, HTG, and control (C) groups.

**Fig 3:**
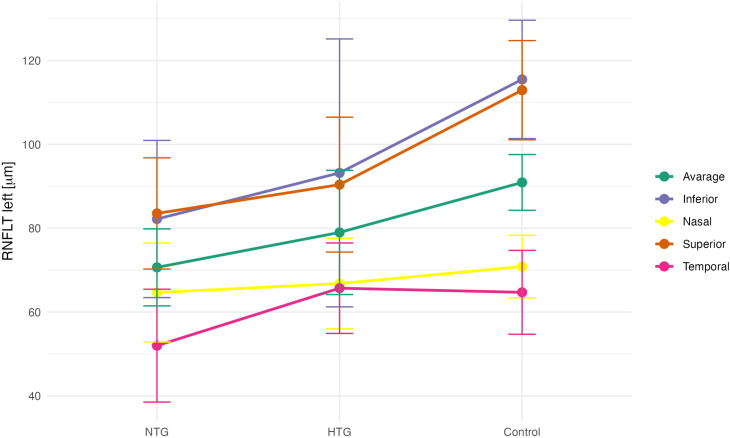
Mean RNFL thickness values (left eye) in the NTG, HTG, and control (C) groups.

To determine whether ophthalmic parameters differed among the NTG, HTG, and control groups, a one-way analysis of variance (ANOVA) was performed. A significant ANOVA result indicates that at least one group differs from the others. In cases of statistical significance, Tukey’s post hoc test was applied to identify which specific group comparisons were significant. The assumptions for parametric testing were met: normal distribution of the variables within each group was assessed using the Shapiro-Wilk test, and homogeneity of variances was evaluated using Levene’s test (Table 4). Group-wise comparisons of mean peripapillary RNFL thickness for both eyes are presented in Figs 2 and 3.

**Table 4:** Comparison of peripapillary RNFL thickness between normal-tension glaucoma (NTG), high-tension glaucoma (HTG), and control (C) groups. Statistically significant results are marked bold.

Parameter	Side	NTG (M ± SD) [µm]	HTG (M ± SD) [µm]	C (M ± SD) [µm]	ANOVA (p-value)	Tukey (p-value)		
						NTG vs C	NTG vs HTG	HTG vs C
**RNFL Average**	right	69.9 ± 8.48	82.3 ± 15.10	83.8 ± 7.84	< 0.0001*	0.0001*	0.013*	0.029*
	left	70.7 ± 9.18	79.0 ± 14.81	90.9 ± 6.65	< 0.0001*	0.0001*	0.0201*	0.1253
**RNFL Superior**	right	85.1 ± 18.17	88.7 ± 17.88	112.2 ± 15.40	0.0003*	0.0004*	0.0059*	0.8672
	left	83.5 ± 13.25	90.4 ± 16.08	112.9 ± 11.83	< 0.0001*	0.001*	0.0008*	0.4371
**RNFL Inferior**	right	72.5 ± 14.22	101.2 ± 22.12	123.2 ± 14.00	< 0.0001*	0.0001*	0.0074*	0.0004*
	left	82.2 ± 18.75	93.2 ± 31.95	115.5 ± 14.11	0.0007*	0.0005*	0.044*	0.4316
**RNFL Temporal**	right	53.4 ± 8.17	69.8 ± 19.63	67.1 ± 11.21	0.0050*	0.0196*	0.0102*	0.8675
	left	52.0 ± 13.46	65.7 ± 10.78	64.7 ± 10.00	0.0062*	0.0155*	0.0178*	0.9772
**RNFL Nasal**	right	70.7 ± 9.18	69.4 ± 14.35	72.3 ± 7.73	0.5670	n.s.	n.s.	n.s.
	left	64.7 ± 11.80	66.8 ± 10.80	70.9 ± 7.46	0.2677	n.s.	n.s.	n.s.

* statistically significant difference (p < 0.05); ANOVA

Four independent measurements of left and right LGN volumes were obtained by two independent observers. For the right LGN, all assumptions for ANOVA were met, and no significant differences between measurements were observed (p = 0.523). For the left LGN, due to non-normality and unequal variances in some datasets, the Kruskal-Wallis test was applied and revealed no significant interobserver differences (p = 0.192). The mean value of the four measurements for each side was used in subsequent analyses, as shown in the Table 5.

**Table 5:** Reproducibility of left and right LGN measurements obtained by four independent observers.

Side	Measurement	Mean Volume [mm^3^]	SD [mm^3^]	Shapiro–Wilk p	Variance Test	Group Comparison Test
Right	1	109.3	14.83	0.2995	Levene’s p = 0.6912	ANOVA: F(3) = 0.75; p = 0.5233
	2	109.6	16.27	0.3288		
	3	106.5	14.71	0.2329		
	4	105.5	11.60	0.5954		
Left	1	100.2	16.98	0.7624	Levene’s p = 0.0068	Kruskal-Wallis: Chi² = 4.74; p = 0.1915
	2	100.8	16.58	0.0562		
	3	94.5	14.12	0.3384		
	4	94.9	12.56	0.0299		

**Fig 4:**
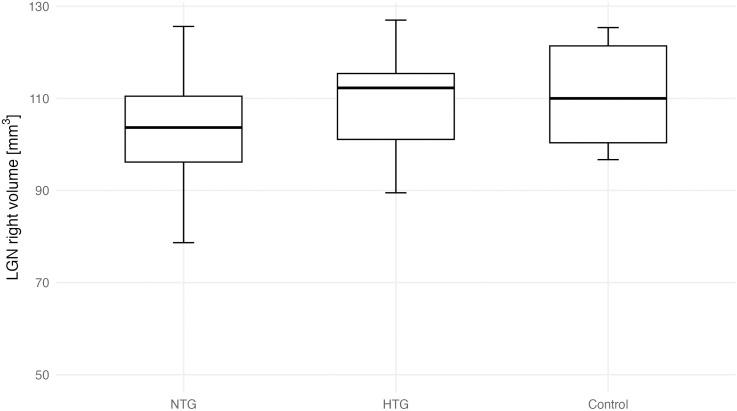
Right LGN volume across groups (NTG, HTG, Control). Boxes show median and IQR; whiskers indicate 1.5 × IQR.

**Fig 5:**
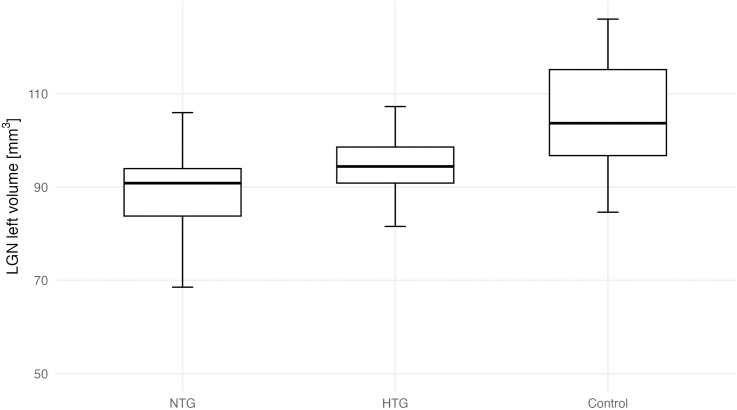
Left LGN volume across groups (NTG, HTG, Control). Boxes show median and IQR; whiskers indicate 1.5 × IQR.

**Fig 6:**
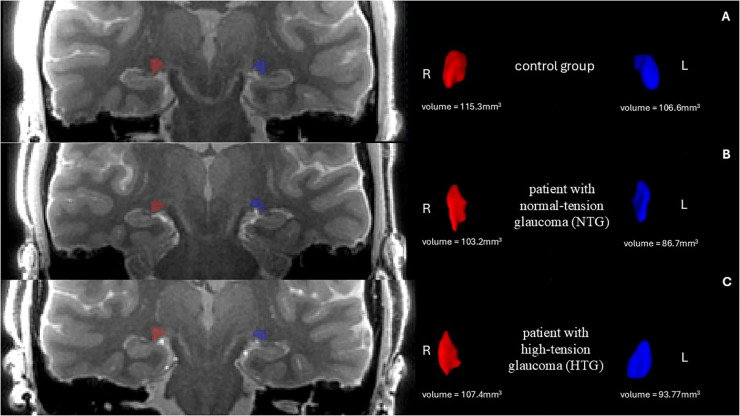
On the left side is the anatomical image of the brain 3D MT-W SILENT with marked LGN, on the right side are 3D view results of LGN manual segmentation using ITK-SNAP. Images show one case chosen from the control group **(A)**, patents with bilateral early-stage normal-tension glaucoma (NTG) (B) and patients with bilateral early-stage high-tension glaucoma (HTG) **(C)**.

A one-way ANOVA was conducted to compare LGN volumes (average, right, and left) between the groups as shown in Table 6. Post hoc Tukey’s comparisons identifying pairwise group differences are presented in Table 7. Assumptions for parametric testing were met. A significant group effect was observed for the left LGN (F(2, 35) = 6.23, p = 0.005) and for the average LGN volume (F(2, 35) = 3.69, p = 0.032), but not for the right LGN (F(2, 35) = 1.45, p = 0.248). Group-wise differences in bilateral LGN volumes are shown in Figs 4 and 5. Effect sizes (η² = 0.174-0.262, 95% CI [0.007-1.000]) indicated moderate group effects. Post hoc Tukey’s tests (Table 7) showed significantly lower left LGN volumes in both NTG (p = 0.007) and HTG (p = 0.031) groups compared with controls, with no significant difference between NTG and HTG (p = 0.935). For the average LGN volume, only NTG differed significantly from controls (p = 0.031). Hedges’ g effect sizes (−0.91 to −1.11) confirmed large magnitude differences for these comparisons. An exploratory asymmetry index ((R–L)/(R + L)) analysis is shown in S2 Fig. Group differences were not statistically significant (estimate ± 95% CI). Representative 3D MT-weighted SILENT MRI images illustrating manual LGN segmentation are shown in Fig 6.

**Table 6:** Comparison of LGN volume between diagnostic groups (NTG, HTG, and controls).

Parameter	Side	NTG (M ± SD)	HTG (M ± SD)	C (M ± SD)	ANOVA		
					statistic	(p-value)	η² (95% CI)
**LGN** **Volume** [mm^3^]	right	103.5 ± 13.85	108.6 ± 11.69	111.0 ± 10.68	F(2,35) = 1.45	0.2481	0.077 [0.000, 1.000]
	left	91.4 ± 12.05	93.1 ± 8.96	106.0 ± 13.26	F(2,35) = 6.23	0.0049*	0.262 [0.060, 1.000]
	av.	97.3 ± 12.20	100.9 ± 8.55	108.5 ± 11.63	F(2,35) = 3.69	0.0323*	0.174 [0.007, 1.000]

* Significance reflects FDR -adjusted p-values for left and right LGN , with p < 0.05 considered statistically significant

**Table 7:** Post-hoc comparisons of LGN volume between groups with mean differences and effect sizes (95% CI).

	Tukey (p)	Δ mean [95% CI]	Hedges’ g [95% CI]
**LGN**			
NTG-C	0.031*	−11.2 [−21.5, −0.9]	−0.91 [−1.67, −0.14]
HTG-C	0.228	−7.6 [−18.7, 3.5]	−0.70 [−1.49, 0.11]
NTG-HTG	0.729	−3.6 [−15.0, 7.9]	−0.32 [−1.11, 0.49]
**LGN right**			
NTG-C	n.s.	−7.7 [−18.9, 3.5]	n.s.
HTG-C	n.s.	−5.4 [−17.8, 7.1]	n.s.
NTG-HTG	n.s.	−2.3 [−14.4, 9.8]	n.s.
**LGN left**			
NTG-C	0.007*	−14.6 [−25.6, −3.6]	−1.11 [−1.89, −0.32]
HTG-C	0.031*	−12.9 [−24.8, −1.0]	−1.06 [−1.88, −0.22]
NTG-HTG	0.935	−1.7 [−14.0, 10.5]	−0.15 [−0.95, 0.64]

* statistically significant difference (p < 0.05)

**Fig 7:**
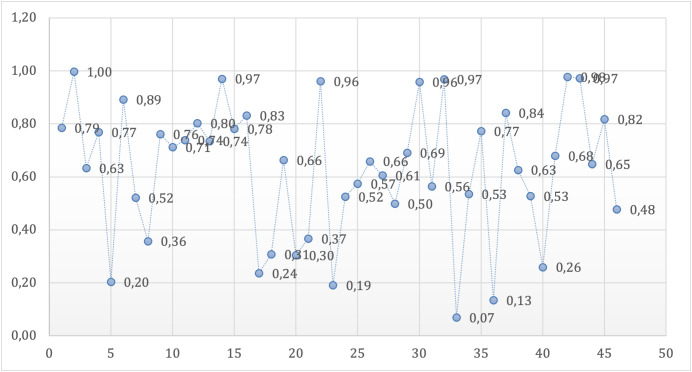
Distribution of ANOVA p-values for left and right hemisphere brain regions. Each point represents the p-value obtained from a one-way ANOVA comparing volumes of a specific brain region among NTG, HTG, and control groups. Regions are ordered according to their appearance in Table 8.

Although statistical comparisons did not reveal significant group differences in the analyzed various brain regions, isolated correlation values approached the threshold for significance, indicating potential trends that warrant further investigation in studies with larger sample sizes or refined methodological approaches as shown in Table 8. One-way ANOVA p-values for brain regions in both hemispheres are presented in Fig 7.

**Table 8:** Comparison of the volumes of various brain regions between NTG, HTG and control groups.

Parameter	F	p-value	Parameter	F	p-value
brain.volume	0.24	0.7850	optic-chiasm	0.00	0.9977
lh_white.matter	0.46	0.6335	rh_white.matter	0.26	0.7691
lh_thalamus	1.67	0.2037	rh_thalamus	0.66	0.5214
lh_hippocampus	0.11	0.8921	rh_hippocampus	1.06	0.3577
lh.BA17 (V1)	0.27	0.7613	rh.BA17 (V1)	0.22	0.8028
lh.BA18 (V2)	0.34	0.7127	rh.BA18 (V2)	0.31	0.7353
lh.BA19 (V5/MT)	0.31	0.7388	rh.BA19 (V5/MT)	0.03	0.9707
lh_caudalmiddle.frontal	0.25	0.7816	rh_caudalmiddlefrontal	0.58	0.5645
lh_cuneus	0.18	0.8323	rh_cuneus	0.03	0.9683
lh_entorhinal	1.50	0.2369	rh_entorhinal	2.87	0.0698
lh_fusiform	1.22	0.3082	rh_fusiform	0.64	0.5350
lh_inferiorparietal	0.42	0.6632	rh_inferior.parietal	0.26	0.7726
lh_inferior.temporal	1.23	0.3047	rh_inferiortemporal	2.13	0.1344
lh_lateraloccipital	1.03	0.3671	rh_lateraloccipital	0.17	0.8416
lh_lingual	0.04	0.9615	rh_lingual	0.47	0.6264
lh_middletemporal	1.74	0.1912	rh_middletemporal	0.65	0.5279
lh_parahippocampal	0.66	0.5248	rh_parahippocampal	1.40	0.2597
lh_postcentral	0.56	0.5738	rh_postcentral	0.39	0.6793
lh_posteriorcingulate	0.42	0.6591	rh_posteriorcingulate	0.02	0.978
lh_precuneus	0.51	0.6052	rh_precuneus	0.03	0.9721
lh_superiorparietal	0.71	0.4992	rh_superiorparietal	0.44	0.6484
lh_superior.Temporal	0.37	0.6910	rh_superiortemporal	0.20	0.8177
lh_insula	0.04	0.9591	rh_insula	0.75	0.4777

* All p-values remained non-significant after FDR correction (p_FDR = 0.9977 for all comparisons)

Pearson correlation analysis revealed no significant associations between left LGN volume and RNFL parameters when analyzed within individual groups. However, when all participants were analyzed together, moderate positive correlations were found between left LGN volume and average, superior, and inferior RNFL thicknesses (p < 0.01). No significant correlations were observed for the right LGN. The results of the Pearson correlation analysis between LGN volume and RNFL parameters are summarized in Table 9.

**Table 9:** Pearson correlation coefficients between LGN volume and peripapillary RNFL thickness in diagnostic subgroups.

Characteristic	N	left side	p-value	N	right side	p-value
		r 95% CI			r 95% CI	
**RNFLT Average**	**37**	**0.54 (0.26, 0.73)**	0.0006*	**37**	**0.20 (−0.14, 0.49)**	**0.2443**
NTG	13	0.15 (−0.44, 0.65)	0.6218	13	0.32 (−0.28, 0.74)	0.287
HTG	10	0.60 (−0.06, 0.89)	0.0698	10	−026 (−0.76, 0.44)	0.4688
Control	14	0.26 (−0.31, 0.69)	0.3708	14	−0.10 (−0.69, 0.45)	0.7257
**RNFLT Superior**	**37**	**0.59 (0.33, 00.77)**	0.0001*	**37**	**0.21 (−0.12, 0.50)**	**0.215**
NTG	13	0.50 (−0.08, 0.82)	0.0852	13	0.38 (−0.22, 0.77)	0.2019
HTG	10	0.50 (−0.18, 0.86)	0.1371	10	−0.11 (−0.69, 0.56)	0.7624
Control	14	0.09 (−0.46, 0.59)	0.7588	14	−0.21 (−0.67, 0.36)	0.468
**RNFLT Inferior**	**37**	**0.44 (0.13, 00.67)**	0.0071*	**37**	**0.24 (−0.09, 0.53)**	**0.1473**
NTG	13	−0.14 (−0.64, 0.44)	0.6382	13	0.07 (−0.50, 0.60)	0.8277
HTG	10	0.54 (−0.13, 0.87)	0.1053	10	−0.04 (−0.65, 0.61)	0.9173
Control	14	0.24 (−0.33, 0.69)	0.4022	14	−0.03 (−0.55, 0.51)	0.9271
**RNFLT Temporal**	**37**	**0.26 (−0.07, 0.54)**	**0.1203**	**37**	**0.12 (−0.21, 0.43)**	**0.4759**
NTG	13	−0.05 (−0.59, 0.51)	0.8677	13	0.34 (−0.26, 0.75)	0.2539
HTG	10	0.33 (−0.37, 0.80)	0.3452	10	−0.36 (−0.81, 0.35)	0.3054
Control	14	0.31 (−0.26, 0.72)	0.2813	14	0.14 (−0.43, 0.62)	0.6441
**RNFLT Nasal**	**37**	**0.30 (−0.03, 0.57)**	**0.0749**	**37**	**−0.11 (−0.42, 0.23)**	**0.5291**
NTG	13	0.19 (−0.41, 0.67)	0.5415	13	−0.04 (−0.58, 0.52)	0.8861
HTG	10	0.57 (−0.09, 0.88)	0.0854	10	−0.39 (−0.82, 0.32)	0.2694
Control	14	−0.04 (−0.57, 0.49)	0.8672	14	−0.07 (−0.58, 0.48)	0.8259

* statistically significant difference (p < 0.05);

Pearson correlation coefficients (*r*) and corresponding *p*-values between LGN volume (left and right hemispheres) and RNFL thickness (average, superior, inferior, temporal, nasal) are presented separately for the full cohort (FULL), normal-tension glaucoma (NTG), high-tension glaucoma (HTG), and control (C) groups, RNFL - retinal nerve fiber layer; LGN - lateral geniculate nucleus.

## 4. Discussion

This study assessed the volumes of the LGN and selected brain regions in patients with early bilateral GON due to primary open-angle glaucoma classified into HTG and NTG based on maximal IOP. To ensure result consistency, only patients with the same glaucoma stage in both eyes were included. High-resolution imaging allowed for precise structural assessment. 7T MRI is primarily used in research, requiring further advancements for clinical integration. This study contributes by refining imaging techniques to enhance its diagnostic applicability in neurodegenerative assessment.

LGN, the primary relay center for RGCs axons, is not merely a transfer station but plays a role in cognition and perception [24,25]. It processes visual information through distinct magnocellular, parvocellular, and koniocellular pathways, with axons projecting to the visual cortex [26]. Our findings confirm previous reports [25,27–29] that LGN volume decreases in glaucoma, correlating with RNFL thickness, suggesting that glaucomatous damage extends to central visual structures. Neuropathological studies have revealed degenerative changes in the LGN, including neuronal shrinkage, cell loss [26,30], and reactive astrogliosis [31] or glial activation [32]. While IOP is a key risk factor, susceptibility to optic nerve damage varies, with some patients developing glaucoma despite normal IOP, and others remaining unaffected despite prolonged elevation. Research has debated whether certain RGCs subtypes are more vulnerable, with some studies suggesting that larger RGCs are most susceptible to IOP-related injury [33]. Functional MRI findings indicate selective impairment of magnocellular LGN layers in early glaucoma [34], supporting the notion of specific neuronal vulnerability. However, no significant LGN volume differences were observed between HTG and NTG in early glaucoma, challenging the hypothesis of primary LGN insufficiency in NTG. This may be attributed to the fact that a loss of 20% to 35% [35–37], and in some cases up to 50% [38], of RGCs is required for visual field changes to become statistically detectable through automated perimetry. As a result, both study groups exhibit significant baseline nerve fiber loss. Detecting differences between early NTG and HTG might require the inclusion of preperimetric glaucoma patients. However, this approach presents challenges related to patient selection and ensuring comparability between study groups.

Epidemiological studies on the glaucoma-AD relationship have yielded conflicting results. Some report increased glaucoma prevalence in AD patients [39–41], while others find no association [42,43]. Meta-analyses suggest glaucoma as an independent risk factor for dementia and AD [44,45]. Studies in animal models of chronic HTG have shown AD-like pathology in the LGN [46], and MRI findings in Primary Open Angle Galucoma (POAG) suggest neurodegenerative processes [47]. Glaucoma shares pathophysiological mechanisms with neurodegenerative diseases, such as RGCs loss and abnormal protein deposition in specific brain regions (e.g., hippocampus) [48]. Studies suggest glaucoma patients exhibit cognitive decline similar to AD [11]. Chronic high IOP has been linked to increased amyloid beta and phospho-tau expression in the hippocampus, potentially contributing to cognitive impairment [46]. These findings support the classification of POAG as a neurodegenerative disease [8], with some considering glaucoma an “ocular AD” [49] or referring to AD as “cerebral glaucoma” [50]. Glaucoma affects not only the visual system but also other brain regions. Pathological changes have been observed in the visual cortex, precuneus, lingual gyrus, insula, and frontal gyrus [51]. Decreased fusiform gyrus (FUG) thickness and reduced fMRI activation were reported in advanced glaucoma [51–55] but not in NTG. Similar FUG changes are also seen in early AD and mild cognitive impairment [56–58]. Selective neuronal vulnerability (SNV), a hallmark of neurodegenerative diseases, may also play a role in glaucoma [59,60]. For example, hippocampal and cortical neuron loss in AD correlates with memory deficits [61].

Our study did not reveal statistically significant differences in brain structures, however, certain trends were observed in the case of the EC, and inferior temporal cortex (ITC) volume between glaucoma subgroups. Compared to age-matched controls, entorhinal cortex (EC) volume was higher in NTG but lower in HTG. As a key relay for sensory information projecting to the hippocampus [62], EC is among the earliest regions affected in AD, exhibiting SNV, neurofibrillary tangles, and neuronal loss. Imaging studies suggest that impaired EC neuronal activity precedes neurodegeneration in AD [55]. Observed alterations in the EC in glaucoma, such as volume asymmetry in NTG patients, highlight the need for further investigation to clarify their potential implications. The ITC, a large visual processing area responsible for object and scene recognition [63], showed thicker right-hemisphere ITC in early HTG compared to NTG, while on the left side, ITC was thinner than in controls. Voxel-wise analyses have previously demonstrated decreased functional connectivity in the right inferior temporal cortex of POAG patients [64], indicating possible structural variations.

Despite differences in non-visual cortical regions, the primary and secondary visual cortices (V1, V2, V3) showed no significant differences between glaucoma groups. Prior studies reported V2 morphometry reductions in advanced POAG [51]. Our previous research found lower BA18 cortical thickness in early NTG compared to controls [65], consistent with Yu et al. findings of bilateral cortical thinning in the anterior visual cortex (BA17, BA18) of POAG patients [53]. Additionally, blood flow reductions in early visual cortical areas have been reported in mild-to-moderate POAG [52]. Similar to our findings, Li et al. [66] noted that structural changes in these regions were absent in early POAG but became pronounced in advanced disease.

This study has several limitations. The sample size was modest, which may reduce statistical power. A slightly lower VFI in the NTG group compared with HTG, despite early disease stage, may result from the greater weighting of central visual field defects in the VFI algorithm. Central corneal thickness, axial length, detailed refractive data, and systemic vascular factors such as hypotension or migraine were not assessed and could have influenced the results. Although LGN segmentation was performed independently by two blinded radiologists, minor variability cannot be excluded. The cross-sectional design also limits causal interpretation. With the current sample (NTG n = 15, HTG n = 10, controls n = 15), power calculations indicate that, at α = 0.05 and 1-β = 0.80, the study was powered to detect standardized between-group differences in LGN volume of approximately d ≈ 1.1 (large effect size). Consequently, while the sample is adequate for identifying large LGN effects, more subtle differences-particularly in cortical regions-may have remained undetected. The non-significant cortical findings should therefore be interpreted cautiously, as a Type II error cannot be excluded. Larger, prospective studies with complete ocular, vascular, and biometric data are needed to confirm these findings.

## 5. Conclusion

Although the present study revealed reduced volumes of the LGN in both NTG and HTG, these correlations did not reach statistical significance between two groups. The observed tendencies may reflect early central involvement in glaucoma, however, the results should be interpreted cautiously. Subtle changes in regions such as the EC and FUG could indicate early or secondary alterations within non-visual networks, but current data are insufficient to establish any direct association with neurodegenerative diseases such as Alzheimer’s disease. Further studies with larger cohorts and multimodal approaches are needed to verify these preliminary observations.

## Supporting information

S1 Table Unadjusted estimates of LGN volume differences between groups. (PDF)

S2 Fig LGN asymmetry index plot. (PNG)
