# Supplementary material for: Comparison of volumes of brain areas in patients with bilateral early high-tension and normal-tension glaucoma in 7 Tesla MRI
Source: PLoS One. 2026 Jan 23;21(1):e0341306. doi: 10.1371/journal.pone.0341306 (PMC12829933; doi:10.1371/journal.pone.0341306)
Supplement: S1 Table — (PDF) [file pone.0341306.s001.pdf]

	NTG	HTG	C	ANOVA		
	(M ± SD)	(M ± SD)	(M ± SD)	**statistic**	**(p-value)**	**η² (95% CI)**
**LGN**	97.3 ± 12.20	100.9± 8.55	108.5 ± 11.63	F(2,35) = 3.69	0.0323*	0.174 [0.007, 1.000]
right	103.5 ± 13.85	108.6 ± 11.69	111.0± 10.68	F(2,35) = 1.45	0.2481	0.077 [0.000, 1.000]
left	91.4 ± 12.05	93.1 ± 8.96	106.0 ± 13.26	F(2,35) = 6.23	0.0049*	0.262 [0.060, 1.000]
