# Supplementary figures and images for: Comparison of volumes of brain areas in patients with bilateral early high-tension and normal-tension glaucoma in 7 Tesla MRI
Source: PLoS One. 2026 Jan 23;21(1):e0341306. doi: 10.1371/journal.pone.0341306 (PMC12829933; doi:10.1371/journal.pone.0341306)

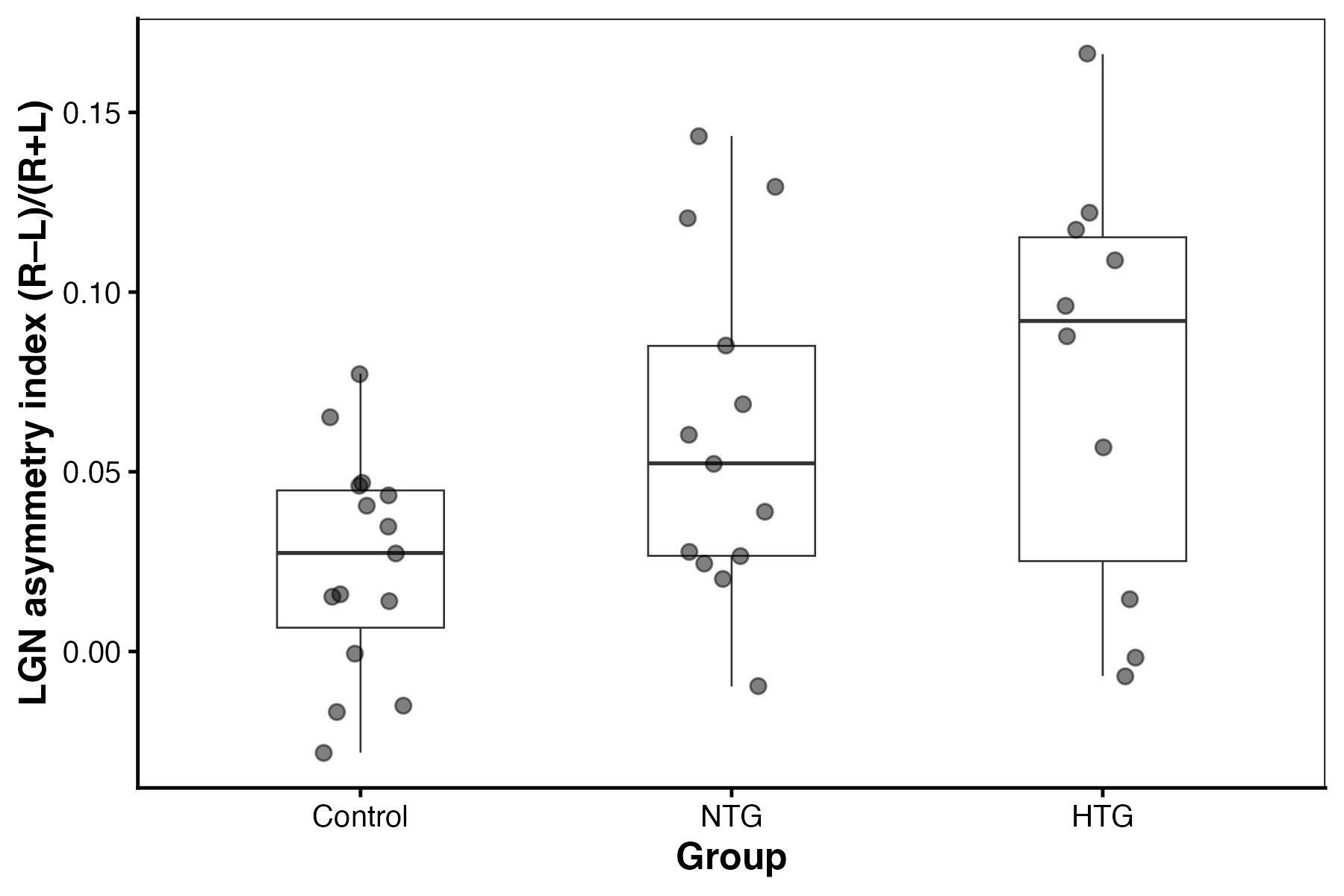

Supplement: S2 Fig — (PNG) [file pone.0341306.s002.png]
